# Prognostic Value of Carbohydrate Antigen 19‐9 and the Surgical Margin in Extrahepatic Cholangiocarcinoma

**DOI:** 10.1002/ags3.12525

**Published:** 2021-11-09

**Authors:** Ryusei Yamamoto, Teiichi Sugiura, Ryo Ashida, Katsuhisa Ohgi, Mihoko Yamada, Shimpei Otsuka, Katsuhiko Uesaka

**Affiliations:** ^1^ Division of Hepato‐Biliary‐Pancreatic Surgery Shizuoka Cancer Center Shizuoka Japan

**Keywords:** carbohydrate antigen 19‐9, extrahepatic cholangiocarcinoma, surgical margin

## Abstract

**Aim:**

The prognostic value of the perioperative carbohydrate antigen 19‐9 (CA19‐9) value and the prognostic relationship between the CA19‐9 value and the surgical margin in extrahepatic cholangiocarcinoma (EHCC) have not been fully discussed.

**Methods:**

A total of 390 patients who underwent curative resection for EHCC between 2002 and 2018 were retrospectively analyzed. According to the perioperative CA19‐9 value, patients were divided into three groups: preoperative normal (Normal, n = 178), preoperative high and postoperative normal (Normalization, n = 155), and preoperative high and postoperative high (Nonnormalization, n = 57). Survival was analyzed according to the perioperative CA19‐9 value and surgical margin.

**Results:**

The optimal cutoff value of CA19‐9 was 37 U/mL. Overall survival (OS) was significantly stratified according to the perioperative CA19‐9 value. The 5‐y OS rates in the Normal, Normalization, and Nonnormalization groups were 53%, 38%, and 23%, respectively (*P* < .001). Although the locoregional recurrence rate was comparable among the groups, the Normal group exhibited distant recurrence less frequently in comparison to the other groups. In the Normal group, the margin status had a significant impact on the OS (surgical resection with a negative margin [R0], 59% vs a microscopically positive margin [R1], 7% at 5‐y, *P* < .001). In contrast, in the Normalization and Nonnormalization groups, the OS rate of the R0 and R1 resection groups did not differ to a statistically significant extent.

**Conclusion:**

The perioperative CA19‐9 value was related to the prognosis of resectable EHCC. A preoperative CA19‐9 value of ≥37 U/mL reflected systemic disease. R0 resection did not affect the survival in this patient group.

## Graphical abstract

The preoperative CA19‐9 value of ≥37 U/mL reflected a systemic disease status. In this patient group, R0 resection did not affect the survival.

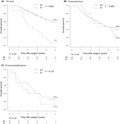

## 1 INTRODUCTION

Surgical resection with a negative margin (R0) has been regarded as the key to achieving long‐term survival with extrahepatic cholangiocarcinoma (EHCC).^1^, ^2^ However, the prognostic impact of R0 resection is sometimes limited in advanced EHCC, such as in cases with lymph node metastasis or abnormally high carbohydrate antigen 19‐9 (CA19‐9) values.^3^, ^4^, ^5^

Given the anatomical complexity of the biliary system, locally advanced EHCC often requires extended surgery to achieve R0 resection, including trisectionectomy,^6^ combined vascular resection and reconstruction (VR),^7^, ^8^ and hepatopancreatoduodenectomy (HPD).^9^, ^10^, ^11^ However, these extensive surgeries are associated with high morbidity and mortality rates.^10^, ^11^, ^12^, ^13^ Thus, high‐risk surgeries should be performed for patients who are expected to benefit from R0 resection. However, the significance of R0 resection has not been fully discussed. Although it is difficult to predict the presence of lymph node metastasis in the preoperative setting,^14^ the CA19‐9 value can be evaluated easily. Therefore, if the CA19‐9 value could be used to predict the prognosis of EHCC, it might help avoid overly invasive surgery.

Abnormally high CA19‐9 values have been reported to be a prognostic factor in resectable EHCC, and to imply the systemic disease status.^15^, ^16^, ^17^, ^18^ Recently, the perioperative change of CA19‐9 has been regarded as an important marker in biliary tract cancer; however, its prognostic value in EHCC has not been sufficiently assessed.^5^, ^19^, ^20^, ^21^ The prognostic relationship between CA19‐9 and the surgical margin remains unclear. A few studies have briefly reported an association between the perioperative CA19‐9 value and the surgical margin in biliary tract cancer^5^, ^19^, ^21^; however, no studies have investigated this association in detail in a population limited to patients with EHCC.

The present study thoroughly investigated survival according to the perioperative CA19‐9 value in patients who underwent resection of EHCC with curative intent. Furthermore, the prognostic value of R0 resection was assessed in groups of patients stratified according to the perioperative CA19‐9 value, in order to reveal the significance of radical resection.

## 2 METHODS

This was a retrospective review of a prospectively maintained EHCC database. Patients who underwent resection for pathologically confirmed EHCC at Shizuoka Cancer Center between September 2002 and December 2018 were reviewed. To avoid the influence of jaundice on CA19‐9, patients in whom the total bilirubin value was >2.0 mg/dL at the time of CA19‐9 measurement were excluded.^5^ Patients who exhibited CA19‐9 values of <2 U/mL were excluded because they were judged to be nonsecretors of CA19‐9 (lack of Lewis antigen glycosyl transferase).^19^, ^22^ Patients whose perioperative CA19‐9 values were not measured were also excluded. Biliary drainage was routinely performed for patients with jaundice, mainly via an endoscopic approach. Portal vein embolization was performed when the future liver remnant was judged to be insufficient.^23^ Neoadjuvant treatment was not performed. Adjuvant treatment was only performed in exceptional cases. Patients who participated in the BCAT trial^24^ received adjuvant gemcitabine chemotherapy, and those who participated in the ASCOT trial^25^ received adjuvant S‐1 chemotherapy. Some patients who exhibited a microscopically positive margin (R1) at the hepatic bile duct received 5‐FU or S‐1 chemotherapy and radiotherapy; the decision on the administration of adjuvant chemoradiotherapy was left to the patient, after providing them with sufficient information to give their informed consent.^26^ This study was approved by our Institutional Ethics Committee (approval number J2020‐87‐2020‐1‐3).

### 2.1 Evaluation of CA19‐9

The preoperative CA19‐9 value was usually measured within 2 weeks before the day of surgery, after the resolution of jaundice and cholangitis. The postoperative CA19‐9 value was usually measured at 2 weeks after the day of discharge. The institutional cutoff value of CA19‐9 was 37 U/mL, according to the standard reference value.^27^ In the present study, the cutoff values of CA19‐9 were determined by a minimum *P*‐value analysis,^28^ which was performed to identify the preoperative and postoperative CA19‐9 values that were associated with the best overall survival (OS).

Eligible patients were divided to three groups according to their perioperative CA19‐9 value: preoperative normal (Normal group), preoperative high and postoperative normal (Normalization group), and preoperative high and postoperative high (Nonnormalization group).

### 2.2 Surgery and pathology

The standard surgical procedure in the author's institution was hepatectomy with extrahepatic bile duct resection for perihilar cholangiocarcinoma and pancreatoduodenectomy for distal cholangiocarcinoma.^29^ The regional lymph nodes were dissected in all patients. Paraaortic lymph node sampling was performed, but surgical resection was typically performed if the intraoperative frozen section diagnosis yielded a positive result. When necessary, HPD and/or VR were aggressively performed to achieve R0 resection. Postoperative complications were graded according to the Clavien–Dindo classification.^30^

Pathological examinations were performed in accordance with the International Union Against Cancer (UICC) TNM classification 7th edition.^31^ In the present study, carcinoma in situ at the ductal margin was defined as R0, because it did not affect OS.^1^

### 2.3 Postoperative follow‐up

The median follow‐up period of the censored patients was 47 mo in the present study. The site of recurrence was confirmed based on radiologic or histologic evidence. Locoregional recurrence was specifically defined as a local ill‐defined mass at the site of choledochojejunostomy, the hepatic artery, or the portal vein, accompanied by positive positron emission tomography findings, increased tumor marker levels, and an increase in size over time on serial imaging performed to detect disease progression.^26^

### 2.4 Statistical analyses

Continuous data were described as the median and interquartile range and were compared using the Mann–Whitney *U*‐test. Categorical variables were compared using Fisher's exact test. The cutoff values of continuous variables according to OS were determined based on a minimum *P*‐value analysis.^28^ Survival curves were generated using the Kaplan–Meier method, and differences were compared by a log‐rank test. A Cox proportional hazards model, with stepwise backward‐forward selection, was used for a multivariate analysis. Two‐sided *P* < .05 were considered statistically significant. All statistical analyses were performed using the R software program (v. 4.0.3; The R Foundation for Statistical Computing, Vienna, Austria).

## 3 RESULTS

In all, 491 consecutive patients underwent resection for EHCC with curative intent, including 272 patients with perihilar cholangiocarcinoma and 219 patients with distal cholangiocarcinoma. A total of 101 patients were excluded for the following reasons (some overlapped): preoperative CA19‐9 value not recorded (n = 4); total bilirubin ≥2.0 mg/dL at the measurement of the CA19‐9 value (n = 68); CA19‐9 < 2 U/mL (n = 19); and postoperative CA19‐9 value not recorded (n = 20). After applying the exclusion criteria, 390 patients were included in the present study.

**FIGURE 1:**
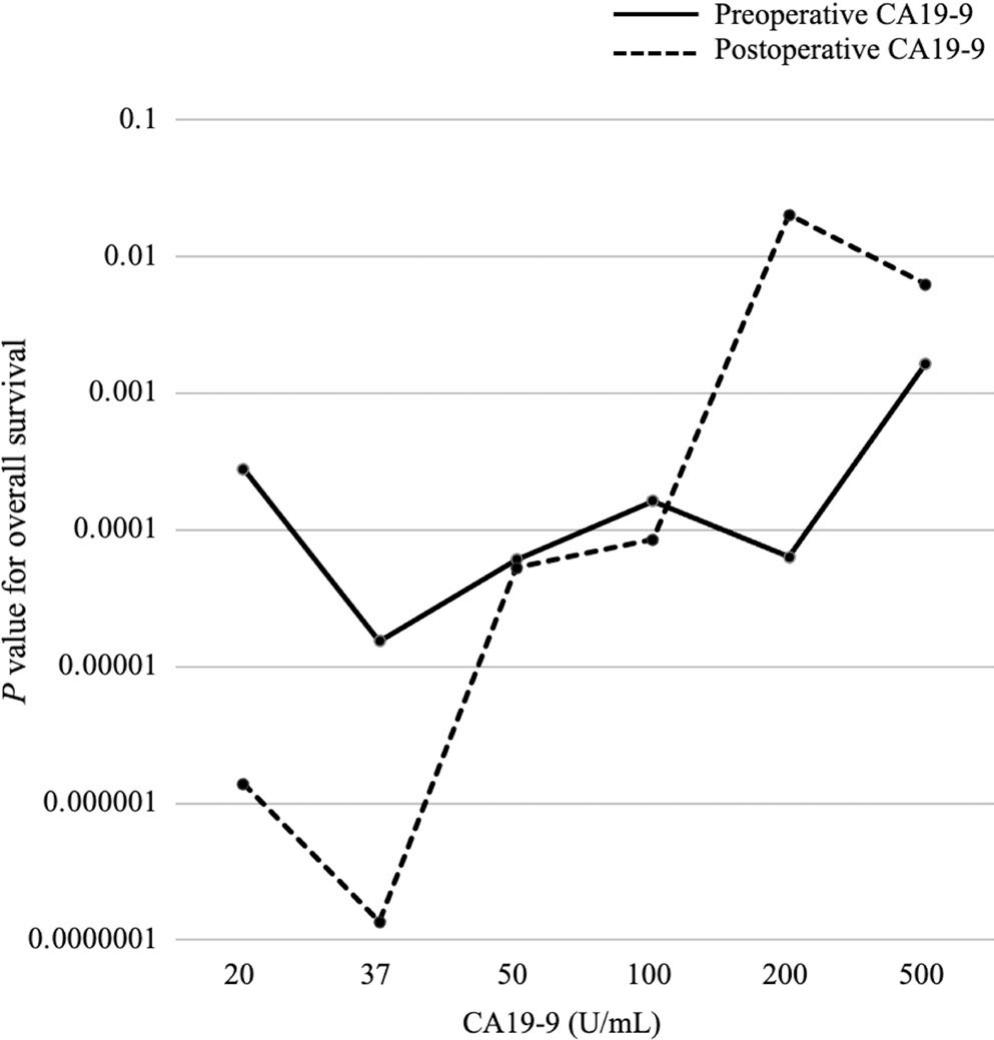
The optimal cutoff value of preoperative and postoperative CA19‐9 for overall survival was determined to be 37 U/mL. CA19‐9, carbohydrate antigen 19‐9

In the minimum *P*‐value analysis, the optimal cutoff value of preoperative and postoperative CA19‐9 was determined to be 37 U/mL; this was the same as the standard cutoff value (Figure 1). The patients were grouped according to their perioperative CA19‐9 values as follows: Normal group, n = 178 (46%); Normalized group, n = 155 (40%); and Nonnormalized group, n = 57 (15%).

Table 1 shows the patient characteristics according to the perioperative CA19‐9 values. The median preoperative CA19‐9 values of the Normal, Normalization, and Nonnormalization groups were 16, 104, and 311 U/mL, respectively, and each difference was statistically significant. The median postoperative CA19‐9 values of the Normal, Normalization, and Nonnormalization groups were 10, 13, and 75 U/mL, respectively, and each difference was statistically significant. Distal cholangiocarcinoma was more frequently observed in the Normal group in comparison to the other groups. The R0 resection rates of the Normal, Normalization, and Nonnormalization groups were 90%, 87%, and 79%, respectively. R1 at the ductal margin was observed in 4%, 7%, and 9% of cases, respectively. R1 at the radial margin was observed in 6%, 8%, and 12% of cases, respectively. Two patients in the Normalization group had R1 at both the ductal and radial margins.

**TABLE 1:** Clinicopathologic characteristics according to the perioperative CA19‐9 value

	(A) Normal	(B) Normalization	(C) Nonnormalization	*P* ^d^		
	(n = 178)	(n = 155)	(n = 57)	A vs B	A vs C	B vs C
Age (y)^a^	70 (66–75)	71 (65–75)	72 (65–77)	.958^e^	.702^e^	.686^e^
Sex ratio, M:F	128:50	111:40	46:11	1	.226	.218
Preoperative CA19‐9 (U/mL)^a^	16 (9–22)	104 (61–266)	311 (103–1211)	<.001^e^	<.001^e^	<.001^e^
Postoperative CA19‐9 (U/mL)^a^	10 (6–17)	13 (8–21)	75 (46–125)	.024^e^	<.001^e^	<.001^e^
Preoperative CEA (ng/mL)^a^	2.0 (1.4–3.0)	2.4 (1.6–3.8)	3.7 (2.4–6.2)	.003^e^	<.001^e^	<.001^e^
Postoperative CEA (ng/mL)^a^	1.9 (1.4–2.6)	1.9 (1.4–2.8)	3.1 (2.2–4.2)	.772^e^	<.001^e^	<.001^e^
Location				<.001	<.001	.227
Perihilar	91 (51)	108 (70)	45 (79)			
Distal	87 (49)	47 (30)	12 (21)			
Biliary drainage	130 (73)	113 (73)	39 (68)	1	.502	.606
Albumin (g/dL)^a^	4.3 (4.0–4.3)	4.0 (3.7–4.2)	3.8 (3.6–4.1)	.117^e^	.007^e^	.109^e^
Total bilirubin (mg/dL)^a^	0.7 (0.5–1.0)	0.9 (0.7–1.3)	0.8 (0.6–1.2)	<.001^e^	.080^e^	.330^e^
C‐reactive protein (mg/dL)^a^	0.20 (0.08–0.52)	0.52 (0.16–1.41)	0.52 (0.18–1.04)	<.001^e^	.004^e^	.638^e^
Operative procedure				<.001	<.001	.107
Hepatectomy	66 (37)	94 (61)	34 (60)			
Pancreatoduodenectomy	70 (39)	37 (24)	8 (14)			
Hepatopancreatoduodenectomy	42 (24)	24 (15)	15 (26)			
Vascular resection	34 (19)	51 (33)	18 (32)	.005	.066	1
Blood transfusion	38 (21)	41 (27)	17 (30)	.303	.210	.608
Complication, grade ≥3^b^	106 (60)	80 (52)	28 (49)	.152	.171	.759
Hospital stay (day)^a^	26 (19–39)	25 (18–38)	25 (17–43)	.621^e^	.906^e^	.738^e^
Histology, G2/G3	111 (62)	105 (68)	37 (65)	.357	.755	.743
T classification, T3/T4^c^	87 (49)	96 (62)	34 (60)	.020	.173	.754
Lymph node metastasis	58 (33)	73 (47)	32 (56)	.072	.002	.279
Distant metastasis	5 (3)	10 (7)	3 (5)	.121	.406	1
Surgical margin				.563	.057	.253
R0	161 (90)	135 (87)	45 (79)			
R1 at ductal margin	7 (4)	10 (7)	5 (9)			
R1 at radial margin	10 (6)	12 (8)	7 (12)			
Adjuvant treatment	18 (10)	20 (13)	7 (12)	.491	.627	1

Note

Values in parentheses are percentages unless otherwise indicated.

Abbreviations: CA19‐9, carbohydrate antigen; CEA, carcinoembryonic antigen.

^a^ Values are median (interquartile range).

^b^ According to the Clavien–Dindo classification.

^c^ According to the UICC 7th edition.

^d^ Fisher's exact test.

^e^ Mann–Whitney *U*‐test.

**FIGURE 2:**
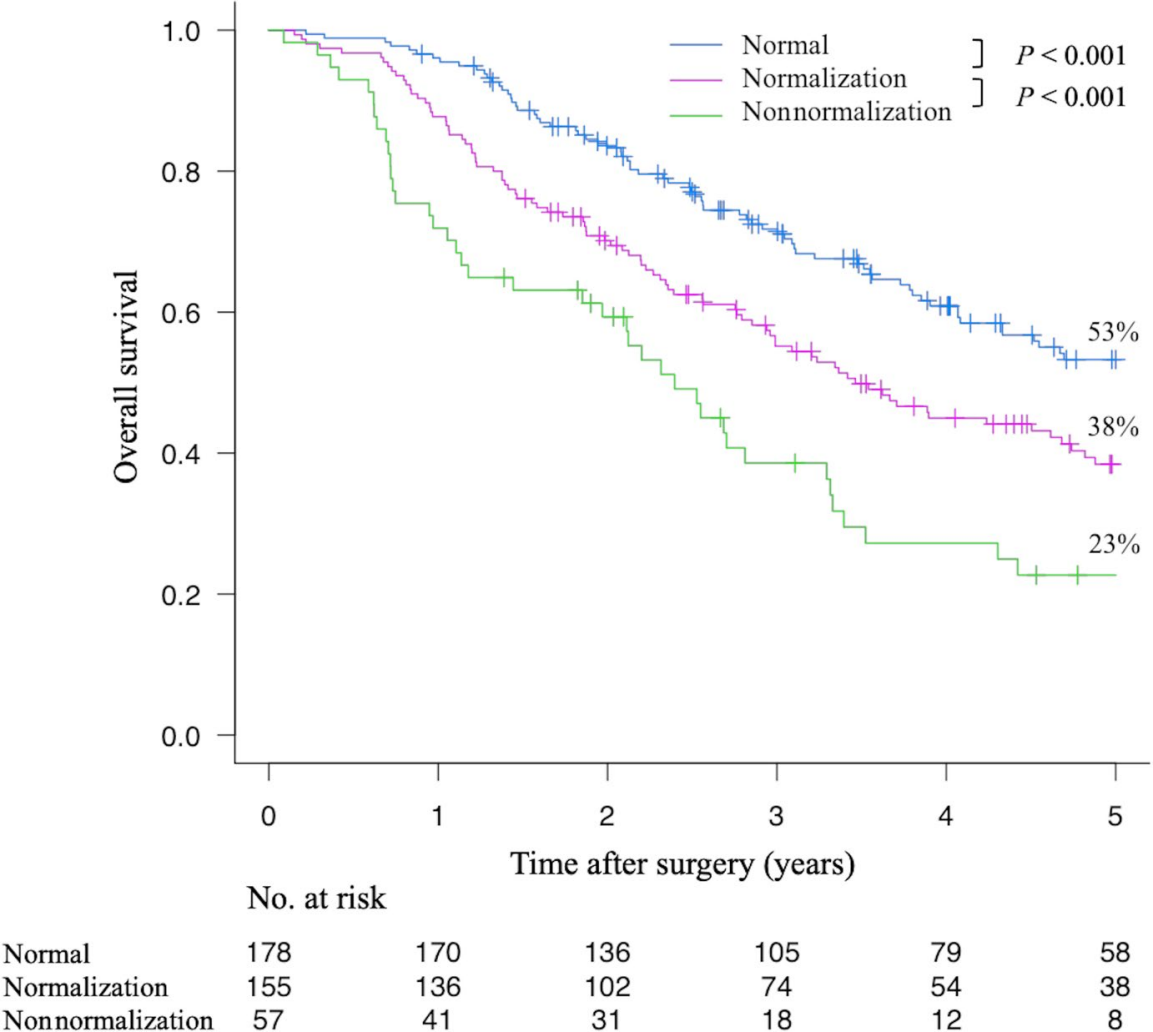
Overall survival according to the perioperative carbohydrate antigen 19‐9 value

Figure 2 shows the OS according to the perioperative CA19‐9 values. Survival was clearly stratified according to the perioperative CA 19‐9 values. The 5‐y OS rates in the Normal, Normalization, and Nonnormalization groups were 53%, 38%, and 23%, respectively, and each difference was statistically significant (*P* < .001). The same tendency was also observed when patients with distant metastasis were excluded from the analysis (Figure S1). Moreover, a similar tendency was observed when patients were divided into perihilar cholangiocarcinoma (Figure S2) and distal cholangiocarcinoma (Figure S3) groups.

Table 2 shows the sites of recurrence according to the perioperative CA19‐9 values. The locoregional recurrence rate did not differ significantly among the groups (Normal group, 15%; Normalization group, 13%; Nonnormalization group, 11%). The frequency of distant recurrence in the Normal group was significantly lower in comparison to the other groups (Normal group, 38%; Normalization group, 54%; Nonnormalization group, 65%). The same tendency was also observed when patients with distant metastasis were excluded (Table S1).

**TABLE 2:** Site of recurrence according to the perioperative CA19‐9 value

	(A) Normal	(B) Normalization	(C) Nonnormalization	*P*		
	(n = 178)	(n = 155)	(n = 57)	A vs B	A vs C	B vs C
Locoregional recurrence	27 (15)	20 (13)	6 (11)	.637	.512	.814
Distant recurrence	67 (38)	84 (54)	37 (65)	.003	<.001	.210
Liver	32 (18)	42 (27)	8 (14)	.049	.550	.067
Retroperitoneal lymph node	20 (11)	16 (10)	14 (25)	.860	.017	.013
Peritoneal	13 (7)	24 (16)	14 (25)	.023	.001	.157
Lung	10 (6)	16 (10)	5 (9)	.151	.368	1
Others	6 (3)	14 (9)	6 (11)	.037	.076	.792

Note

Values in parentheses are percentages.

**FIGURE 3:**
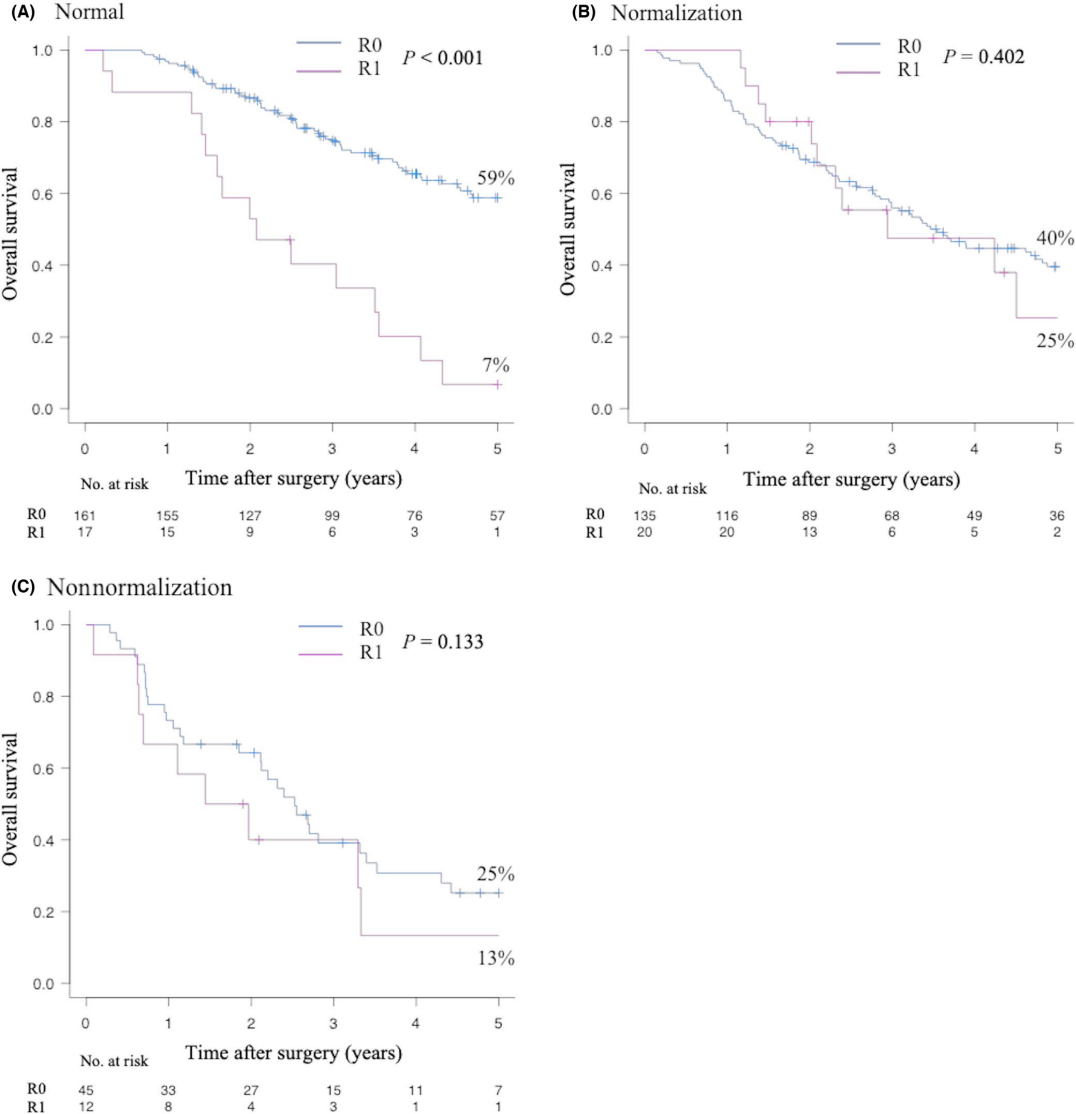
Overall survival according to surgical margin in each group. A: Normal group. B: Normalization group. C: Nonnormalization group

Figure 3 shows the OS according to the surgical margin status in each of the groups. In the Normal group, OS was significantly better in patients who received R0 resection than in those who received R1 resection (59% vs 7% at 5‐y, *P <*.001). In the Normalization and Nonnormalization groups, the OS of the patients who received R0 resection was not significantly different from that in patients who received R1 resection. The same tendency was also observed when patients with distant metastasis were excluded (Figure S1). Moreover, a similar tendency was observed when patients were divided into perihilar cholangiocarcinoma (Figure S2) and distal cholangiocarcinoma (Figure S3) groups.

The multivariate analysis revealed that both the preoperative CA19‐9 value (*P* = .018) and the postoperative CA19‐9 value (*P* < .001) were independently associated with OS (Table 3).

**TABLE 3:** Univariate and multivariate analyses of factors associated with overall survival

Variables	n	OS (%)		*P* ^b^	Multivariate	
		3‐y	5‐y		HR (95% CI)	*P* ^c^
Age (y)						
<75	270	62.6	44.9	.052		
≥75	120	55.4	38.5			
Gender						
Female	105	67.9	56.2	.012		
Male	285	57.4	37.8			
Preoperative CA19‐9 (U/mL)						
<37	178	71.8	53.3	<.001	1.00 (reference)	.015
≥37	212	50.9	34.3		1.42 (1.07–1.89)	
Postoperative CA19‐9 (U/mL)						
<37	322	64.3	47.1	<.001	1.00 (reference)	<.001
≥37	68	41.5	23.5		1.84 (1.34–2.52)	
Preoperative CEA (ng/mL)						
<3.0	250	66.6	48.0	<.001		
≥3.0	140	49.1	33.8			
Postoperative CEA (ng/mL)						
<3.0	287	64.4	45.3	<.001		
≥3.0	47.0	47.0	35.4			
Location						
Perihilar	244	57.1	39.9	.040		
Distal	146	66.1	48.5			
Albumin (g/dL)						
<4.0	186	51.1	33.3	<.001	1.50 (1.15–1.97)	.003
≥4.0	204	68.5	50.1		1.00 (reference)	
Hepatopancreatoduodenectomy						
Absent	308	61.3	42.8	.677		
Present	82	56.5	43.1			
Vascular resection						
Absent	287	66.8	49.4	<.001	1.00 (reference)	<.001
Present	103	41.7	23.8		1.83 (1.37–2.45)	
Blood transfusion						
Absent	294	60.2	41.9	.842		
Present	96	60.6	45.8			
Histologic grade						
G1	137	71.9	54.4	<.001	1.00 (reference)	<.001
G2/G3	253	54.1	36.6		1.76 (1.31–2.36)	
T classification^a^						
T1/T2	173	68.3	51.0	.002		
T3/T4	217	54.1	36.7			
Lymph node metastasis						
Absent	227	70.2	54.7	<.001	1.00 (reference)	<.001
Present	163	46.1	25.5		1.67 (1.26–2.20)	
Distant metastasis						
Absent	372	61.6	44.1	.020		
Present	18	30.8	15.4			
Surgical margin						
R0	341	62.7	46.5	<.001	1.00 (reference)	<.001
R1	49	43.2	15.1		1.89 (1.31–2.74)	
Adjuvant treatment						
Absent	345	59.4	43.2	.913	1.00 (reference)	.029
Present	45	67.9	38.3		0.61 (0.39–0.95)	

Abbreviations: CA19‐9, carbohydrate antigen; CEA, carcinoembryonic antigen; CI, confidence interval; HR, hazard ratio; OS, overall survival.

^a^ According to the UICC 7th edition.

^b^ Log‐rank test.

^c^ Cox hazard test.

## 4 DISCUSSION

The present study showed the utility of the perioperative CA19‐9 value and the prognostic relationship between the perioperative CA19‐9 value and the surgical margin in a relatively large cohort of patients with resectable EHCC. The optimal cutoff value was determined to be 37 U/mL. Patients in whom the preoperative CA19‐9 value was <37 U/mL showed a good prognosis, and greatly benefited from R0 resection. The preoperative CA19‐9 value of ≥37 U/mL reflected the presence of systemic disease. R0 resection did not affect survival in this patient group.

Hepato‐biliary‐pancreatic surgeons have made efforts to achieve complete eradication of EHCC through extended hepatectomy, VR, and HPD, approaches that require highly sophisticated surgical skills and perioperative management approaches.^6^, ^7^, ^8^, ^9^, ^10^, ^11^, ^29^ In the present study, VR and HPD were performed for 103 patients (26%) and 81 patients (21%), respectively. However, some patients did not benefit from R0 resection, despite receiving aggressive surgery, and showed high morbidity and mortality. A few studies reported the prognostic relationship between CA19‐9 and R0 resection in biliary tract cancer among patients with normalized and nonnormalized CA19‐9 values.^5^, ^19^, ^21^ However, the significance of R0 resection in patients with normal CA19‐9 values has not been reported. The present study showed that patients with preoperative CA19‐9 values of <37 U/mL—but not those with values of ≥37 U/mL―benefited from R0 resection.

It is known that CA19‐9 influences the prognosis of biliary tract cancer; the standard cutoff value is 37 U/mL.^5^, ^19^, ^20^, ^27^ However, the optimal cutoff value of CA19‐9 for EHCC remains controversial. Wang et al^18^ reported a cutoff value of 150 U/mL, but their sample size was very limited. Although Lee et al^21^ reported a cutoff value of 300 U/mL, preoperative CA19‐9 >300 U/mL did not remain a significant factor in their multivariate analysis. Based on the statistical analysis of the present study, the cutoff value of 37 U/mL was found to be appropriate. Moreover, the multivariate analysis revealed that preoperative and postoperative CA19‐9 values of ≥37 U/mL were both independent prognostic factors. Therefore, the standard cutoff value of 37 U/mL was found to be the optimal cutoff value for CA19‐9.

Recently, the usefulness of the perioperative change in CA19‐9 in patients with resectable biliary tract cancer has received attention.^5^, ^19^, ^20^, ^21^ It is obvious that the Nonnormalization group would show a dismal prognosis. Yamashita et al^19^ reported that patients with normalized CA19‐9 showed equivalent survival to those with normal CA19‐9. However, two‐thirds of their cohort was composed of patients with intrahepatic cholangiocarcinoma; thus, it may be difficult to directly apply their results to resectable EHCC. Lee et al^21^ reported that among a cohort of patients with resected perihilar cholangiocarcinoma, patients with normal CA19‐9 values and those with normalized CA19‐9 showed similar OS; however, their surgical outcomes seemed to be poor, as the R0 resection rate in patients with normal CA19‐9 values was 62%, while the 5‐y OS rate was only 29%. If the R0 resection rate could be further improved, a prognostic difference might have been observed between patients with normal and normalized CA19‐9 values. In the present study, OS in the Normalization group was significantly poorer in comparison to the Normal group, which was similar to the results reported by Kim et al^20^ The locoregional recurrence rate did not differ among the groups. However, in the Normal group distant recurrence was observed less frequently in comparison to the other groups, while the distant recurrence rate did not differ between the Normalization and Nonnormalization groups. Namely, a high preoperative CA19‐9 value reflects systemic disease in patients with EHCC. In these patients, a satisfactory prognosis could not be achieved by radical resection alone. These results suggest that R0 resection was meaningful in the Normal group but not in the Normalization or Nonnormalization groups.

The present authors would not discourage hepato‐biliary‐pancreatic surgeons from aiming for R0 resection in cases in which the CA19‐9 value is ≥37 U/mL; however, in these cases excessive surgery, such as HPD with VR, should be considered after extremely careful patient selection. Conversely, in patients with CA19‐9 values of <37 U/ml, such procedures should be performed because R0 resection offers the best chance of achieving long‐term survival.

The present study was associated with some limitations, including its retrospective nature and single‐center setting. The results of this study cannot be applied to patients with jaundice or CA19‐9 values of <2 U/mL. The period from surgery to postoperative CA19‐9 measurement differed according to the individual length of postoperative hospital stay, which seemed to affect the postoperative CA19‐9 value. However, this period could not affect the postoperative CA19‐9 value because the half‐life of CA19‐9 is only 0.5 d.^32^ Furthermore, the number of subjects was not sufficient to draw broad interpretations. Thus, a multi‐institutional study with a large cohort should be conducted to validate the findings of the present study.

In conclusion, the perioperative CA19‐9 value was related to the prognosis of resectable EHCC. The preoperative CA19‐9 value of ≥37 U/mL reflected a systemic disease status. R0 resection did not affect survival in this patient group.

## DISCLOSURE

Ethical Approval: The protocol for this research project was approved by a suitable Institutional Ethics Committee and conformed to the provisions of the Declaration of Helsinki. The Institutional Review Board of Shizuoka Cancer Center approved the study (Approval No. J2020‐87‐2020‐1‐3). Informed consent was substituted by the informed opt‐out procedure because of the retrospective nature of the study, and anonymous clinical data were used for the analysis.

Funding: The authors declare that no external funding was received for this study.

Conflict of Interest: The authors declare no conflicts of interest in association with the present study.

## Supporting information

Fig S1

Fig S2

Fig S3

Table S1
