# Supplementary figures and images for: Prognostic Value of Carbohydrate Antigen 19‐9 and the Surgical Margin in Extrahepatic Cholangiocarcinoma
Source: Ann Gastroenterol Surg. 2021 Nov 9;6(2):307–15. doi: 10.1002/ags3.12525 (PMC8889865; doi:10.1002/ags3.12525)

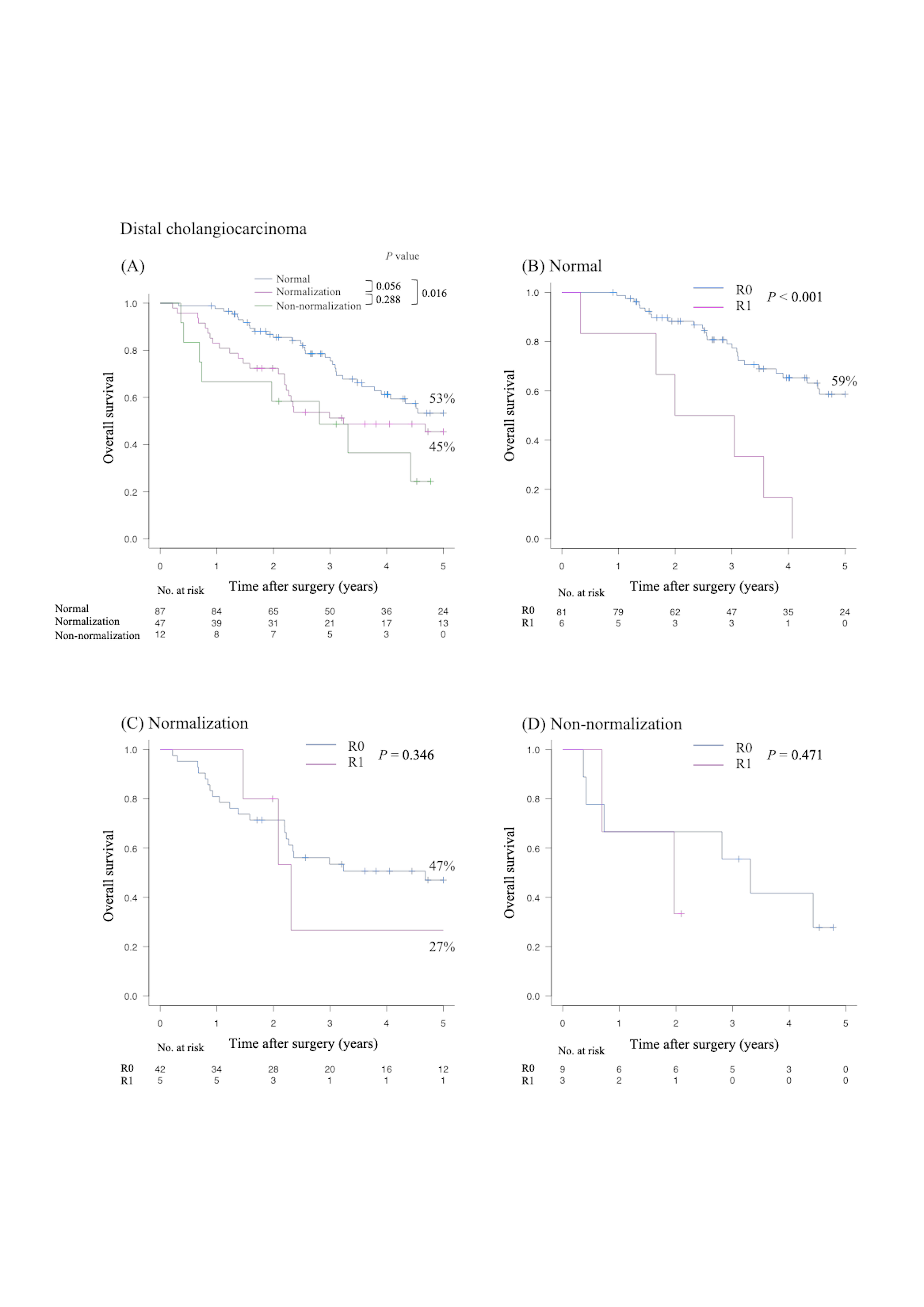

Supplement: Supplementary file 3 — Fig S3 [file AGS3-6-307-s001.tiff]
