# Supplementary material for: Prognostic Value of Carbohydrate Antigen 19‐9 and the Surgical Margin in Extrahepatic Cholangiocarcinoma
Source: Ann Gastroenterol Surg. 2021 Nov 9;6(2):307–15. doi: 10.1002/ags3.12525 (PMC8889865; doi:10.1002/ags3.12525)
Supplement: Supplementary file 4 — Table S1 [file AGS3-6-307-s003.docx]

| **Supplementary Table 1.** Site of recurrence according to the perioperative CA19-9 value with the exclusion of patients with distant metastasis | | | | | | |
| --- | --- | --- | --- | --- | --- | --- |
|  | **(A) Normal** | **(B) Normalization** | **(C) Non-normalization** | ***P*^*^** | | |
|  | (n = 173) | (n = 145) | (n = 54) | A *vs.* B | A *vs.* C | B *vs.* C |
| Locoregional recurrence | 27 (16) | 19 (13) | 6 (11) | 0.632 | 0.511 | 0.813 |
| Distant recurrence | 62 (36) | 75 (52) | 34 (63) | 0.005 | <0.001 | 0.200 |
| Liver | 32 (19) | 36 (25) | 8 (15) | 0.173 | 0.683 | 0.178 |
| Retroperitoneal lymph node | 16 (9) | 15 (10) | 12 (22) | 0.850 | 0.017 | 0.037 |
| Peritoneal | 12 (7) | 23 (16) | 12 (22) | 0.012 | 0.004 | 0.301 |
| Lung | 9 (5) | 12 (8) | 5 (9) | 0.365 | 0.330 | 0.782 |
| Others | 6 (4) | 14 (10) | 6 (11) | 0.035 | 0.039 | 0.793 |
| Values in parentheses are percentages.*Fisher's exact test. | | | | | | |
